# CodY is modulated by YycF and affects biofilm formation in *Staphylococcus aureus*

**DOI:** 10.3389/fmicb.2022.967567

**Published:** 2022-10-11

**Authors:** Shizhou Wu, Boquan Qin, Shu Deng, Yunjie Liu, Hui Zhang, Lei Lei, Guoying Feng

**Affiliations:** ^1^Department of Orthopedics, Orthopaedic Research Institute, West China Hospital, Sichuan University, Chengdu, China; ^2^Boston University Henry M. Goldman School of Dental Medicine, Boston, MA, United States; ^3^West China School of Public Health, Sichuan University, Chengdu, China; ^4^Department of Preventive Dentistry, West China Hospital of Stomatology, Sichuan University, Chengdu, China; ^5^College of Electronics and Information Engineering, Sichuan University, Chengdu, China

**Keywords:** antisense, biofilm formation, *Staphylococcus aureus*, YycFG, CodY

## Abstract

**Background:**

*Staphylococcus aureus* (*S. aureus*) is the leading cause of various infective diseases, including topical soft tissue infections. The goals of this study were to investigate the roles of YycF and CodY in the regulation of biofilm formation and pathogenicity.

**Methods:**

Electrophoretic mobility shift assay (EMSA) was conducted to validate the bound promoter regions of YycF protein. We constructed the *codY* up-regulated or down-regulated *S. aureus* mutants. The biofilm biomass was determined by crystal violet microtiter assay and scanning electron microscopy (SEM). Quantitative RT-PCR analysis was used to detect the transcripts of biofilm-related genes. The live and dead cells of *S. aureus* biofilm were also investigated by confocal laser scanning microscopy (CLSM). We constructed an abscess infection in Sprague Dawley (SD) rat models to determine the effect of CodY on bacterial pathogenicity. We further used the RAW264.7, which were cocultured with *S. aureus*, to evaluate the effect of *CodY* on macrophages apoptosis.

**Result:**

Quantitative RT-PCR analyses reveled that YycF negatively regulates *codY* expression. EMSA assays indicated that YycF protein directly binds to the promoter regions of *codY* gene. Quantitative RT-PCR confirmed the construction of dual- mutant stains *codY* + AS*yycF* and *codY*-AS*yycF*. The SEM results showed that the biofilm formation in the *codY* + AS*yycF* group was sparser than those in the other groups. The crystal violet assays indicated that the *codY* + AS*yycF* group formed less biofilms, which was consistent with the immunofluorescence results of the lowest live cell ration in the *codY* + AS*yycF* group. The expression levels of biofilm-associated *icaA* gene were significantly reduced in the *codY* + strain, indicating *codY* negatively regulates the biofilm formation. Furthermore, CodY impedes the pathogenicity in a rat-infection model. After cocultured with bacteria or 4-h *in vitro*, the apoptosis rates of macrophage cells were lowest in the *codY* + group.

**Conclusions:**

YycF negatively regulate the expression of *codY*. By interaction with *codY*, YycF could modulate *S. aureus* biofilm formation *via* both eDNA- dependent and PIA- dependent pathways, which can be a significant target for antibiofilm. CodY not only impedes the pathogenicity but also has a role on immunoregulation. Thus, the current evidence may provide a supplementary strategy for managing biofilm infections.

## Introduction

*Staphylococcus aureus* (*S. aureus*), a Gram-positive opportunistic pathogen, is the leading cause of various infectious diseases, including topical soft tissue infections, osteomyelitis, and endocarditis (Wertheim et al., 2004). Due to biofilm formation, *S. aureus* infection has become more challenging to treat in recent years (Schilcher and Horswill, 2020). Biofilm formation is responsible for persistent infections, difficult to eradicate, and much more resistant to environmental stimuli. Thus, biofilms are an essential target for infection treatment, and many strategies targeting biofilms have been developed to attenuate the pathogenicity of bacteria. In *S. aureus*, approximately 16 TCSs (Two-component systems) play a role in adaptation to environmental changes. YycFG is the only TCS essential for the viability of bacteria and significantly modulates gene expression, which is associated with biofilm formation and pathogenicity (Villanueva et al., 2018; Jenul and Horswill, 2019; Rapun-Araiz et al., 2020). YycFG TCS, also known as the VicRK/WalRK TCS, consists of the sensor histidine protein kinase YycG and its cognate response regulator YycF. By phosphorylation, YycG activates YycF and controls downstream gene expression (Wang et al., 2021).

YycFG has a major role in controlling biofilm formation in low-G + C Gram-positive bacteria, including *S. aureus* (Dubrac et al., 2007). By directly binding to the promoter region of the *ica* operon, activated YycF can positively trigger extracellular polysaccharide (EPS) synthesis, which is associated with biofilm construction (Xu et al., 2017). The *ica* operon is a chromosomal gene locus that comprises the intercellular adhesion gene *icaA* and regulates the production of polysaccharide intercellular adhesin (PIA). Additionally, YycF can indirectly stimulate the expression of *ica* operon by controlling the expression of the global transcriptional regulator SarA, resulting in biofilm aggregation (Wu et al., 2021b). Hence, the YycFG TCS represents a promising target to modulate *S. aureus* biofilms.

Research has shown that CodY [control of dciA (decoyinine induced operon) Y] is a global repressor regulator in Gram-positive bacteria (Chapeton-Montes et al., 2020). In response to environmental signals such as the amount of branched-chain amino acids (BCAAs) [isoleucine, leucine, and valine (ILV)] and GTP, CodY adjusts metabolism and virulence gene regulation (Pohl et al., 2009). *Via* recognition of a conserved sequence motif (AATTTTCWGAAAATT) (Brinsmade, 2017), CodY competes with RNA polymerase for binding to a promoter and primarily represses the target genes. In *S. aureus*, biofilm development is thought to occur mainly *via* PIA-dependent and PIA-independent biofilm formation pathways. CodY can act as a repressor of *ica* and modulates PIA-dependent biofilm formation (Majerczyk et al., 2008). PIA-independent biofilms are mainly based on the aggregation of extracellular DNA (eDNA) and/or protein. CodY also contributes to PIA-independent formation by repressing the expression of secreted proteases and nucleases (Nuc) (Mlynek et al., 2020). Both eDNA and PIA can work synergistically in biofilm organization.

CodY-targeted biofilm genes have been extensively studied in *S. aureus*, but the regulatory relationship between CodY and YycFG TCS is largely unknown (Augagneur et al., 2020). In this study, we used electrophoretic mobility shift assays (EMSAs) to verify the binding of YycF to *codY* promoters and identify negative regulation of YycF on CodY by RT–PCR to gain insight into the relationship between CodY and YycFG and their coordinating adjustments to *S. aureus* biofilm formation and pathogenicity. We showed that *S. aureus* YycF acts as a repressor to control the activity of CodY, thus contributing to biofilm formation and pathogenesis in infectious diseases.

## Methods and materials

### Bacterial strains and biofilm growth conditions

As previously described, *S. aureus* strain ATCC29213 was cultured in tryptic soy broth (TSB) at 37°C and 5% CO_2_. Briefly, 500 μL of *S. aureus* suspension was inoculated into 10 mL fresh TSB medium to mid-logarithmic phase (optical density at 600 nm [OD_600_] = 0.5), and a log-phase suspension was prepared for further investigation. For biofilm formation, sterilized glass disks (10-mm diameter) were dropped into 24-well microtiter plates and cocultured with log-phased suspension for 24 h.

### Electrophoretic mobility shift assay to detect bound codY promoter regions of YycF protein

We performed electrophoretic mobility shift assays to determine whether the YycF protein could directly bind to the promoter regions of *codY*. To generate YycF His-Tag fusion proteins, pET-22b (Novagen) was applied to yield pET-*yycF* at Huabio Biotech (Hangzhou, China). Then, the above plasmids were transformed into *E. coli* BL21 for recombinant proteins. We isolated recombinant proteins from bacterial suspension culture after a 3-h induction with 1 mM IPTG. The acquired recombinant proteins were purified through affinity chromatography on Ni^2+^ NTA agarose (Qiagen). The purified YycF protein was visualized *via* Coomassie staining after SDS–PAGE.

The PCR amplicon of the *codY* promoter region was generated from the *S. aureus* ATCC29213 genomic DNA sample using primers labeled with the 5′ FAM (Roche) (see Table 1). The amplified DNA fragments were purified according to the manufacturer’s instructions (Tiangen Biotech, Beijing, China). After purification, labeled DNA fragments (0.02 pmol) were incubated with recombinant YycF protein at various concentrations from 0 to 60 pmol. After 30 min of incubation on ice, the samples were loaded on native PAGE gels in 0.5 × TBE buffer (44.5 mM Tris-HCl, 44.5 mM boric acid, 1 mM EDTA, pH 8.0). Native PAGE was prepared with 5 × TBE (445 mM Tris-HCl, 445 mM boric acid, 10 mM EDTA, pH 8.0), 30% Acr-Bis (29:1), 50% glycerinum, 10% ammonium persulfate (APS), and N,N,N′,N′-tetramethylethylenediamine (TEMED). Gel electrophoresis was performed at 110 V for 90 min on ice, according to our previous study (Lei et al., 2018).

**TABLE 1:** Sequences of primers in this study.

Primers	Sequence 5′–3′ (forward/reverse)
**RT-qPCR**	
*icaA*	5′-GATTATGTAATGTGCTTGGA-3′/ 5′-ACTACTGCTGCGTTAATAAT-3′
*yycF*	5′-TGGCGAAAGAAGACATCA-3′/ 5′-AACCCGTTACAAATCCTG-3′
*yycG*	5′-CGGGGCGTTCAAAAGACTTT-3′/ 5′-TCTGAACCTTTGAACACACGT-3′
*icaD*	5′-ATGGTCAAGCCCAGACAGAG-3′/ 5′-CGTGTTTTCAACATTTAATGCAA-3′
*16S rRNA*	5′-GTAGGTGGCAAGCGTTATCC-3′/ 5′-CGCACATCAGCGTCAACA-3′
**EMSA**	
P*codY*	5′-AGTCGATGAGTCTGGGACATAATT-3′/ 5′-TGTGAAATATCAATTTGATTG-3′

### Construction of codY-upregulated or -downregulated *Staphylococcus aureus* mutants

We constructed the *S. aureus yycF* downregulating strain (AS*yycF*) as previously described (Wu et al., 2021a). To investigate the subsequent effects of *codY*, we constructed *codY*-upregulated or -downregulated expression mutants. To downregulate *codY* expression, antisense sequences were applied to construct a *codY*-downregulated expression mutant by transformation of a plasmid expressing antisense *codY* (AS*codY*) into *S. aureus* ATCC29213. AS*codY* was engineered by Sangon Biotech (Shanghai, China) by inserting the antisense sequences of *codY* into restriction sites between *Bam*HI and *Eco*RI. In addition, a *codY*-upregulated expression mutant (*codY* +) was constructed by transformation of the *codY*-encoding sequences inserted into the pDL278 plasmid in ATCC29213.

The methods of construction for dual mutants were modified according to our previous study (Zhang et al., 2022). To generate overexpression strain *codY* + AS*yycF* (*yycF* low-expression and *codY* overexpression mutant), *codY*^–^AS*yycF* (codY low-expression and yycF low-expression mutant), anti-sense sequences of *yycF* were obtained by oligonucleotides synthesis and connected with cody coding region or antisense *codY* cloned into the pDL278 shuttle vector (Sangon Biotech, Shanghai, China), generating recombinant plasmid pDL278 *codY* + AS*yycF* or pDL278 *codY*^–^AS*yycF*.

### Analysis of gene expression using quantitative real-time PCR

To investigate the interactions between *yycF* and *codY* and the effect on biofilm-associated gene expression, quantitative real-time PCR (qRT–PCR) was performed. The *codY^–^yycF*^–^, *codY*^–^, *codY*^+^*yycF*^–^, *codY*^+^, and ATCC29213 (as a control) strains were cultured to the mid-logarithmic phase. Total RNA was extracted and purified from each strain with the MasterPure™ RNA Purification Kit (Epicenter Technologies, Madison, WI, USA). The purified RNA was reverse transcribed to cDNA with the RevertAid First Strand cDNA Synthesis Kit (Thermo Scientific). Quantitative real-time PCR assays were performed with a LightCycler 480 system (Roche, Basel, Switzerland) with the primers listed in Table 1 and the 16S rRNA gene as an internal control. Threshold cycle values (CT) were determined, and the abundance of each gene was expressed relative to that of the 16S rRNA gene. Each sample was analyzed in triplicate, and the data were analyzed according to the 2^–ΔΔCT^ method.

### Crystal violet assay and epifluorescence staining for biofilm biomass

A crystal violet assay was performed to evaluate the biomass of biofilms, including *codY*^–^AS*yycF*, *codY*^–^, *codY*^+^AS*yycF*, *codY*^+^, and ATCC29213 (as a control). After 24 h of culture in TSB medium, the biofilm samples were stained with 0.1% (w/v) crystal violet for 15 min. The dye bound to the biofilms was transferred into a new plate, and the absorbance was measured with a microplate reader (ELX800, Gene) at 595 nm (Wu et al., 2021b). In addition, the biofilms were labeled with SYTO9 and PI for epifluorescence observation. Live strains were stained green, while dead strains appeared red. Three random fields in each specimen were visualized using epifluorescence microscopy (Nikon Eclipse TE-2000S, Melville, NY).

### Characterizing biofilm morphologies

To observe the biofilm structure of each group, scanning electron microscopy (SEM) (Inspect, Hillsboro, OR, USA; SEM) was conducted. The 24 h biofilm samples were washed with PBS twice and fixed with 2.5% glutaraldehyde for 4 h. Then, the biofilm samples were dehydrated and dried in a critical point dryer. After being coated with gold powder, micrographs of the biofilm samples were evaluated.

### Abscess model for evaluation of pathogenicity

The ability of *S. aureus* to form biofilms contributes to major microbial infections. To determine the effect of *codY* on biofilm infection, which can be modulated by YycF, we constructed an abscess infection in Sprague Dawley (SD) rat models. The animal experiments were approved by West China Hospital Animal Welfare Committee (NO. 20220606004). The rats were randomized into four experimental groups: *S. aureus* as a positive control, SSN group, AS*codY*, *codY* + and normal control group (*n* = 5 rats per group). After anesthetization with ketamine (60 μg/g) and xylazine (6 μg/g), we injected 0.1 mL of a bacterial suspension (1.2 × 10^9^CFU/mL) into deep calf muscle and observed after 36 h. A palpable fluctuant mass in the calf muscle was identified for model establishment (Wyss et al., 2004). For histopathological analysis, the muscle tissue was excised and fixed in 10% neutral-buffered formalin for 48 h. Tissue sections were processed and stained with hematoxylin-eosin (HE) according to standard protocols (Cardiff et al., 2014).

### The effect of CodY on macrophages

We also used RAW264.7 cells to evaluate the effect of *CodY* on macrophage apoptosis. RAW264.7 cells were cultured in DMEM supplemented with 10% heat-inactivated fetal bovine serum (FBS). Bacterial suspensions of *S. aureus* ATCC29213, *codY*- and codY + strains at the log phase were diluted to achieve a multiplicity of infection (MOI) of 100:1. The number of *S. aureus* was determined by serial dilution with the plate counting method. Cells were inoculated into a 6-well plate at 3.0 × 10^5^ cells/well. After the cells grew for 12 h and formed a monolayer, 200 μL of *S. aureus* (MOI = 100:1) was added to each well for 4 h and treated with lysostaphin (10 μg/mL) for 12 min to kill extracellular *S. aureus*. To detect RAW264.7 cell apoptosis, we used the Annexin V-FITC/propidium iodide (AV/PI) dual staining AP-101-100-kit (Multisciences, China) to test the apoptosis rate of RAW264.7 cells following the manufacturer’s instructions. Briefly, after coculture with *S. aureus*, the cells were digested with trypsin, collected by centrifugation, washed with PBS, stained with Annexin V-FITC and PI, and analyzed by FCM (Becton CytoFLEX) (Xu et al., 2020). The cell concentration for FCM was modulated to 1.0 × 10^7^/mL. Each sample was added with 5μL Annexin V-FITC and PI for 30 min at 4°C. After centrifugation at 300 g for 5min, the supernatant was removed, resuspended in 500 mL PBS and analyzed for cell apoptosis.

### Data analysis

All statistical data were analyzed in SPSS 16.0 (SPSS Inc., Chicago, IL, USA). The Shapiro–Wilk test was used to analyze the distribution of data, and the Bartlett test was used to determine the homogeneity of variances. For parametric testing, we adopted one-way ANOVA to assess the statistical significance of variables followed by the Tukey test. Differences in the data were considered significant at *P* < 0.05.

## Results

### *YycF* negatively regulates CodY expression

**FIGURE 1:**
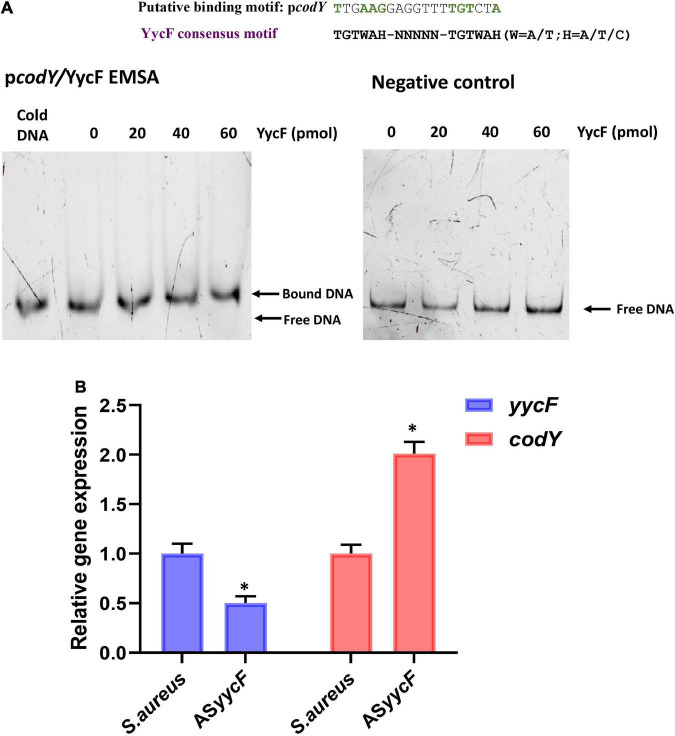
YycF negatively regulates *codY* expression. **(A)** Consensus YycF binding motif and candidate sequences in promoters of *codY*. TGTWAH-NNNNN-TGTWAH, where W is A/T and H is A/T/C; EMSA in which promoter regions were obtained by PCR and FAM-labeled. As the negative control, a DNA fragment the same size as the promoter region and similar AT: GC mole ratio, but missing the YycF consensus binding sequence, was used to rule out non-specific binding. **(B)** Quantitative RT-PCR analysis showed the gene transcripts in *S. aureus*, and AS*yycF* strains. *S. aureus* gene expression was relatively quantified by RT-PCR using 16S as an internal control (*n* = 5, *P < 0.05).

The methods of construction for dual mutants were modified according to our previous study (Zhang et al., 2022). To generate overexpression strain *codY* + AS*yycF* (*yycF* low-expression and *codY* overexpression mutant), *codY*-AS*yycF* (*codY* low-expression and *yycF* low-expression mutant), anti-sense sequences of *yycF* were obtained by oligonucleotides synthesis and connected with *codY* coding region or antisense *codY* cloned into the pDL278 shuttle vector (Sangon Biotech, Shanghai, China), generating recombinant plasmid pDL278 *codY* + AS*yycF* or pDL278 *codY*-AS*yycF*. Quantitative RT–PCR analyses revealed that the transcription of *codY* was elevated in AS*yycF* strains (Figure 1A). To reveal the possible interactions between YycF and candidate targeted genes, EMSA was performed on the promoter regions of the *codY* gene. As demonstrated in Figure 1B, the promoter region of *codY* contained a putative YycF-binding consensus motif. The YycF protein directly binds to the promoter regions of the *codY* gene.

### CodY interaction with YycF affects biofilm morphology

**FIGURE 2:**
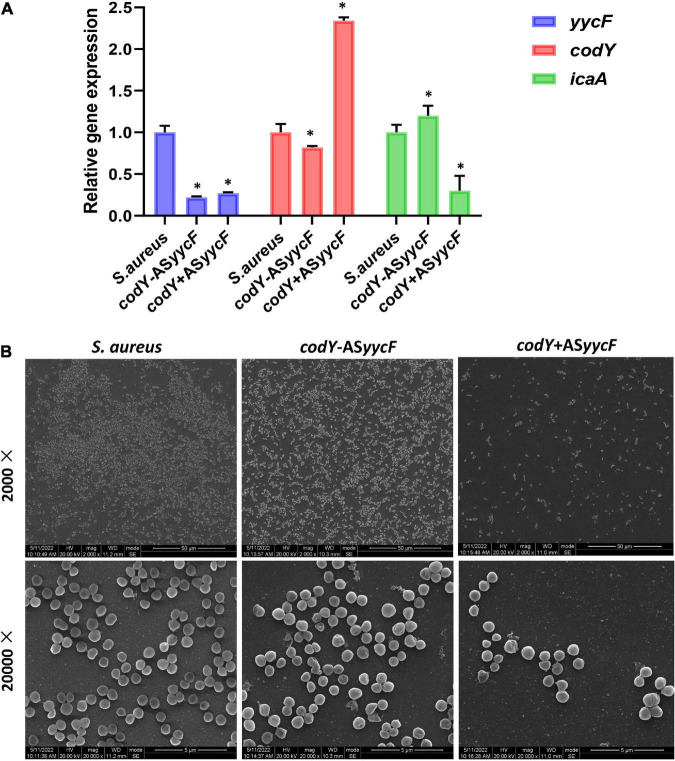
CodY interaction with *yycF* affects biofilm morphology. **(A)** Quantitative RT-PCR analysis showed the gene expressions in *S. aureus*, *codY* + AS*yycF*, and *codY*-AS*yycF* dual- mutant stains. *S. aureus* gene expression was relatively quantified by RT-PCR using 16S as an internal control (*n* = 5, *P < 0.05). **(B)** SEM images of *S. aureus*, *codY* + AS*yycF*, and *codY*-AS*yycF* dual- mutant stains.

**FIGURE 3:**
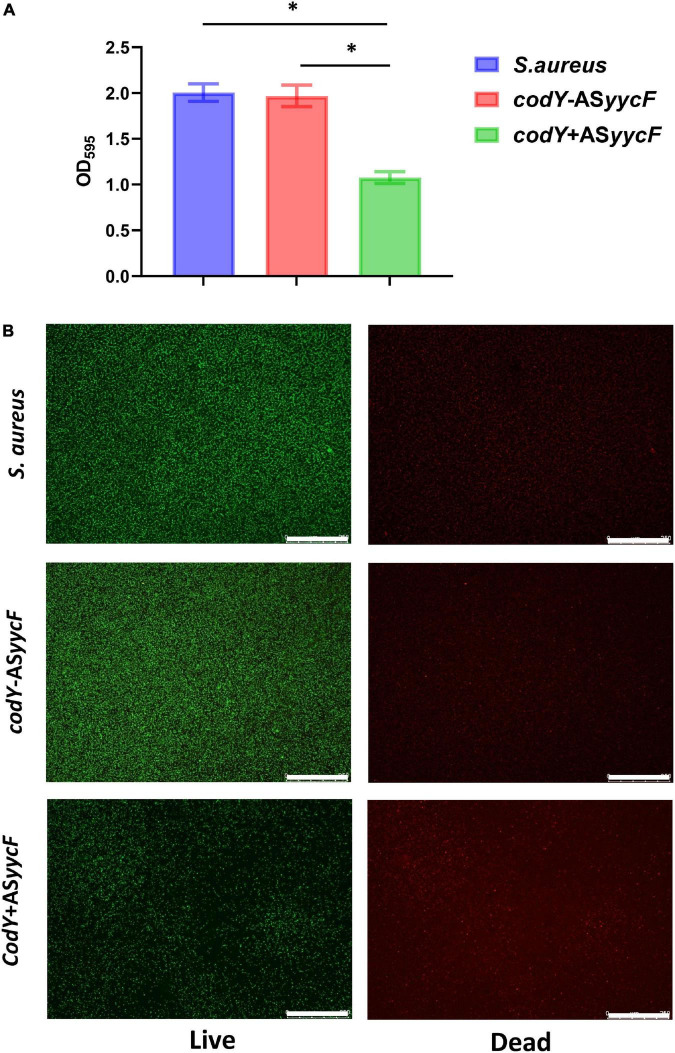
CodY interaction with *yycF* regulates the biofilm biomass. **(A)** Biomass of *S. aureus*, *codY* + AS*yycF*, and *codY*-AS*yycF* dual- mutant stains were quantified by crystal violet staining. Optical densities at 595 nm were measured (*n* = 5, *P < 0.05). **(B)** The CLSM observations of *S. aureus*, *codY* + AS*yycF* and *codY*-AS*yycF* dual- mutant stains. Green, viable bacteria (SYTO 9); red, dead bacteria (PI); scale bars, 250 μm.

Quantitative RT–PCR demonstrated that in the dual-mutant stains *codY* + AS*yycF* and *codY*-AS*yycF*, the expression levels of *yycF* genes were significantly reduced. Furthermore, the expression levels of the biofilm-associated *icaA* gene were significantly reduced in the *codY* + AS*yycF* strain compared with the *S. aureus* and *codY*-AS*yycF* strains (*P* < 0.05; Figure 2A), which can be attributed to the reduced biomass of the *codY* + AS*yycF* strain. The SEM results showed that the biofilm formation in the *codY* + AS*yycF* group was sparser than those in the other groups (Figure 2B), and that *codY* interacted with *yycF* in regulating biofilm formation. Quantitatively, we evaluated the ability of the *S. aureus* strains to form biofilms in the TSB culture. The biomass was quantified *via* the crystal violet assay, and the *codY* + AS*yycF* group formed fewer biofilms than the *S. aureus* group, as demonstrated by the reduction in OD_595_ values from 1.9 to 1.0 (Figure 3A). Similarly, the immunofluorescence density of the live cells in the *codY* + AS*yycF* group was the lowest compared with the *S. aureus* and *codY*-AS*yycF* groups (Figure 3B).

### CodY suppressed biofilm morphology and biofilm-associated genes

**FIGURE 4:**
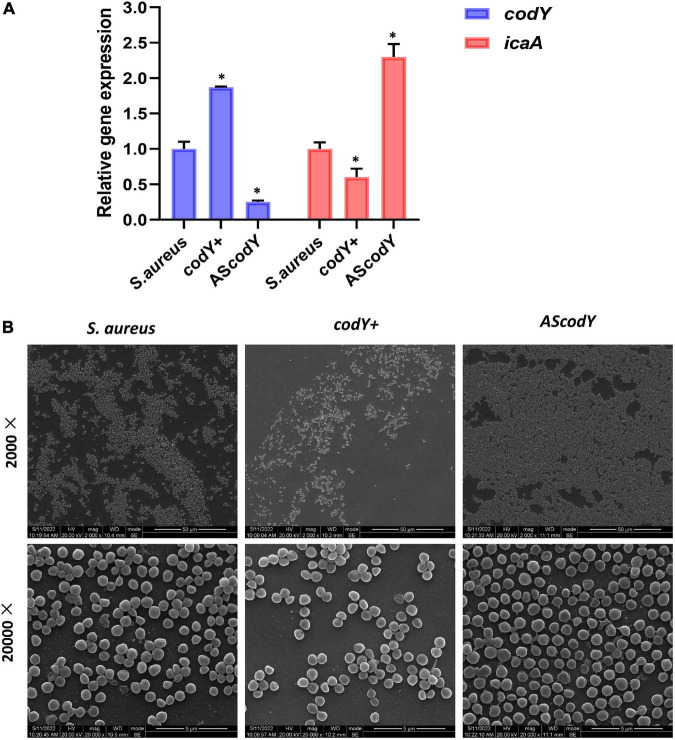
CodY suppressed biofilm morphology and biofilm-associated genes. **(A)** Quantitative RT-PCR analysis showed the gene expressions in *S. aureus*, *codY* + and *codY*- mutant stains. *S. aureus* gene expression was relatively quantified by RT-PCR using 16S as an internal control (*n* = 5, *P < 0.05). **(B)** SEM images of *S. aureus*, *codY* + and *codY*- mutant stains.

**FIGURE 5:**
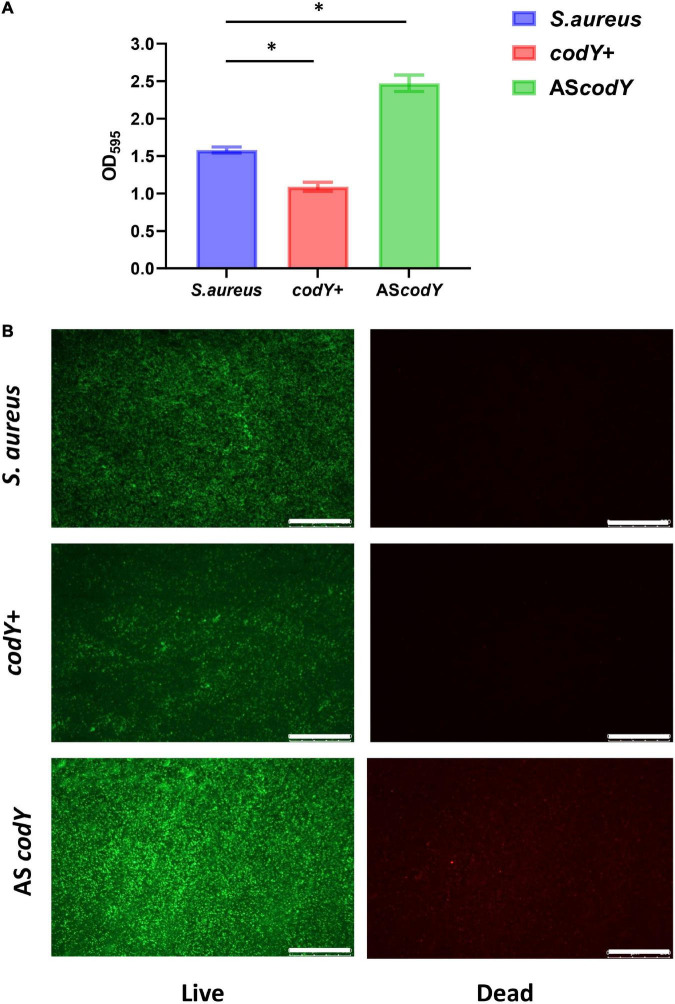
CodY decreases the biomass of *S. aureus* biofilm**. (A)** Biomass of *S. aureus*, *codY* + and *codY*- mutant stains were quantified by crystal violet staining. Optical densities at 595 nm were measured (*n* = 5, *P < 0.05). **(B)** The CLSM observations of *S. aureus*, *codY* + and *codY*- mutant stains. Green, viable bacteria (SYTO 9); red, dead bacteria (PI); scale bars, 250 μm.

To further explore the potential roles of the *codY* gene in biofilm formation, we constructed the *codY* + strain (*codY* overexpression strain) and AS*codY* strain (*codY* low-expression strain). Quantitative RT–PCR analyses demonstrated the construction of the *codY* + strain and AS*codY* strain (Figure 4A, blue column). Furthermore, the expression levels of the biofilm-associated *icaA* gene were significantly reduced in the *codY* + strain but increased in the AS*codY* strain (*P* < 0.05; Figure 4A, red column), indicating that *codY* negatively regulates biofilm-associated genes. The SEM results showed that the biofilm formation in the *codY* + group was sparser than that in the other groups (Figure 4B). In particular, the AS*codY* strains presented dense biofilms. The biomass was quantitively measured by crystal violet staining (Figure 5A). The *codY* + group formed the lowest biomass, while the AS*codY* strain presented the highest biomass, as demonstrated by the reduction in OD_595_ values from 2.5 to 1.0 (Figure 5A). Accordingly, the immunofluorescence density of the live cells in the *codY* + group was the lowest compared with the *S. aureus* and AS*codY* groups (Figure 5B).

### CodY impeded pathogenicity in a rat infection model

**FIGURE 6:**
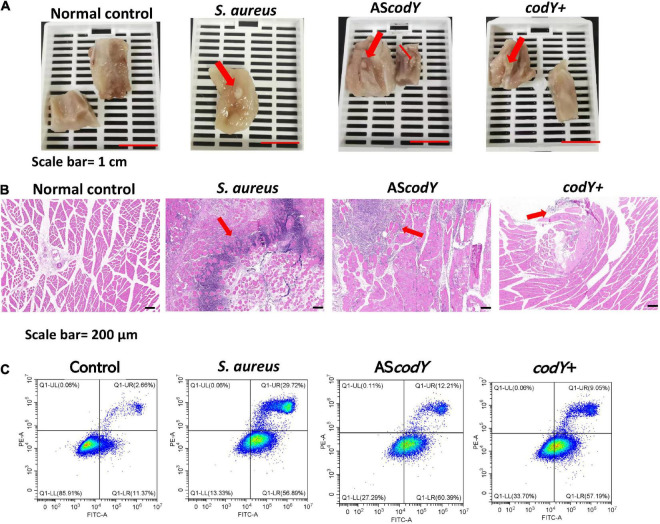
CodY impedes pathogenicity in a rat-infection model. **(A)** Gross observation of the abscess in *S. aureus*, *codY* + and *codY*- infected group; scale bars, 1 cm. **(B)** HE staining of the abscess lesions in *S. aureus*, *codY* + and *codY*- infected group; scale bars, 200 μm. **(C)** The apoptosis rates of macrophage cells when co-cultured with *S. aureus*, *codY* + and *codY*- respectively were measured by the flow cytometer.

Thirty-six hours after muscle injection of *S. aureus*, *codY*+, and AS*codY* strains, the rats were sacrificed by euthanasia under deep anesthesia (ketamine/xylazine) by cervical dislocation. The infection sites were dissected under macroscopic observation. Among all groups, infectious lesions with diameters of approximately 3 mm and 1.5 mm were observed in the *S. aureus* and AS*codY* groups, respectively. However, in the codY-overexpressing group (c*odY* +), the abscess in muscle was obscure, and there were only unhealthy tissues with a diameter of less than 2 mm (Figure 6A). Correspondingly, the percentage of inflammatory cell infiltration was measured by ImageJ, and there were approximately 13% inflammatory cells in the *S. aureus* group. The percentages of inflammatory cell infiltration were approximately 9 and 6% for the AS*codY* and c*odY* + groups, respectively (Figure 6B). After coculture with bacteria for 4 h *in vitro*, the apoptosis rates of macrophages were measured by flow cytometry. The total apoptosis rate in *S. aureus* (ATCC29213) was 86.61%. In the AS*codY* group, the total apoptosis rate was 72.60%, which was higher than that of 66.24% in the *codY* + group (Figure 6C).

## Discussion

*Staphylococcus aureus* is a major human pathogen that is responsible for a wide range of infectious diseases. The propensity of bacteria to form biofilms is one of most crucial factors contributing to pathogenesis and resistance (McCarthy et al., 2015). In *S. aureus*, biofilm organization is thought to occur mainly *via* two mechanisms, polysaccharide intercellular adhesin (PIA)-based and eDNA/protein-based pathways. Both pathways contribute to the construction of a self-produced extracellular matrix, which is primarily comprised of exopolysaccharides, proteins, and extracellular DNA (eDNA) for cell-to-cell or cell-to-host attachment. The potential mechanisms of biofilm formation are critical for developing strategies to control biofilms and biofilm-related infections.

PIA synthesis is modulated by the *ica* locus. According to a previous study, the expression of *ica* is positively controlled by YycFG, which is the only essential TCS in *S. aureus* that regulates bacterial metabolism, including virulence and biofilm formation (Clausen et al., 2003; Wu et al., 2019b). YycFG is reported to modulate *Staphylococcus epidermidis* biofilm formation in an *ica*-dependent manner (Xu et al., 2017). In *Bacillus subtilis*, YycFG is directly involved in regulation of cell wall synthesis and modification (Wu et al., 2019). The YycG protein acts as a sensor to respond to environmental signals, and YycF can directly regulate different sets of vital functional genes by binding to promoter regions (Winkler and Hoch, 2008). The putative recognition sequence of YycF is composed of two hexanucleotide direct repeats separated by five nucleotides [5′-TGT(A/T)A(A/T/C)-N5-TGT(A/T)A(A/T/C)-3′]. However, CodY, as a global regulator, can negatively regulate *ica* expression and inhibit biofilm formation. In *Clostridium difficile*, the variability of CodY-dependent regulation is an important contributor to the bacterial virulence and sporulation (Nawrocki et al., 2016). In *Bacillus subtilis*, CodY can be seen to regulate the entire protein utilization pathway (Barbieri et al., 2015). Additionally, in major gram-positive pathogens, several virulence factors are regulated by CodY (Stenz et al., 2011). To identify the interaction of YycF and *codY*, we analyzed the promoter of *codY*, and the consensus motif of YycF was found (Figure 1A). The EMSA results revealed that YycF can bind to the promoter of *codY* and potentially regulate its expression (Figure 1A).

The mutation in *yycF* reduced biofilm formation and led to decreased transcripts in the *ica* operon (Howden et al., 2011). However, our RT–qPCR assays showed downregulation in *yycF* combined with subsequent elevated expression in *codY* (Figure 1B). Thus, we speculated that YycF negatively modulated the expression of *codY*. To further explore the interactions of YycF with *codY*, we constructed dual mutants expressing *codY* and *yycF* (Figure 2). In the *codY-*AS*yycF group* (indicating that both *codY* and *yycF* expression were downregulated), the PCR results indicated that the expression of *codY* decreased while *ica* expression significantly increased. Consistent with previous work, *ica* (PIA synthesis) is negatively regulated by CodY (Majerczyk et al., 2008, 2010). With the relatively higher *ica* expression in the *codY-*AS*yycF* group, the biofilm biomass increased (Figures 2B, 3A). Whereas the *codY* + AS*yycF* group presented lower *ica* expression, the biofilm biomass significantly decreased (Figures 2B, 3A).

In addition, YycFG TCS (also known as WalRK, VicRK, and MicAB TCS) plays a central role in bacterial viability (Haag and Bagnoli, 2017; Villanueva et al., 2018). In the *codY* + AS*yycF* group, the density of strains was significantly downregulated (Figure 3). However, both *yycF* and *codY* expression decreased in the *codY*-AS*yycF* group, and the accumulation of strains was similar to that in the *S. aureus* group (Figure 3). Therefore, the downregulation of *yycF* can inhibit *S. aureus* viability, and this viability alteration can be partially complemented by repressing *codY*. CodY, as a global transcription factor, typically represses gene expression and regulates physiology for growth and survival under various levels of nutrient depletion (King et al., 2018). YycF as an essential and global regulator responds to various physiological metabolic processes in *S. aureus* (Wu et al., 2021a). Therefore, multiple reasons including eDNA- dependent and PIA- dependent pathways as well as slow growth rate and reduced viability will inhibit biofilm formation. In addition to CodY/YycF regulated eDNA- dependent and PIA- dependent pathways, we also found reduced viability in Figures 3, 5 and slow growth rate in Supplementary Material.

**FIGURE 7:**
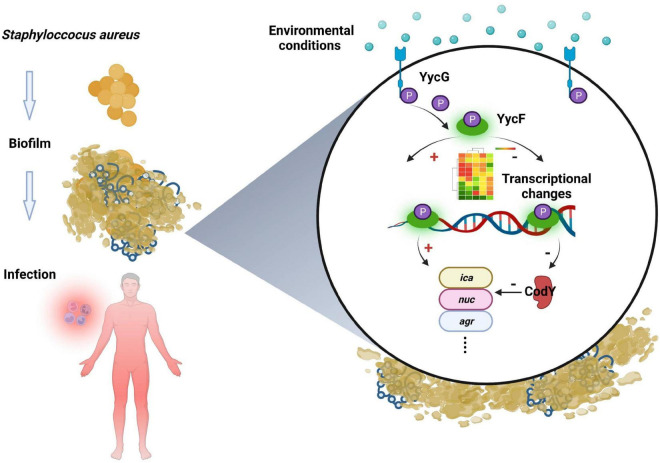
Working model of CodY interactions with YycF affects biofilm formation in *Staphylococcus aureus.*

In addition to bacterial growth and biofilm formation, YycF also regulates the expression of genes involved in cell wall metabolism and virulence (Bleul et al., 2021). Subsequently, to observe the specific effect of *codY* on biofilm organization, which can be modulated by YycF, we constructed *codY* overexpression (*codY* +) and downregulation (*codY*-) mutants. The decrease in CodY activity promotes cell aggregation and biofilm formation (Brinsmade, 2017). Our SEM result in Figure 4B indicated a significant increase in the AS*codY* biofilm, which is consistent with this conclusion. By utilizing available eDNA and PIA, CodY demonstrates a synergistic effect combining a DNA-dependent strategy with a PIA-based strategy for biofilm formation (Mlynek et al., 2020). Considering the present findings, YycF negatively modulates *codY* expression while positively participating in an eDNA/PIA dual-dependent manner for biofilm organization in *S. aureus* (Figure 7).

In *S. epidermidis*, YycF is bound to the promoter of *ica* and increases *ica* expression (Xu et al., 2017). Similarly, YycF was shown to interact with the *ica* promoter region and contribute to PIA-based biofilms in our previous study (Wu et al., 2021b). In the present study, YycF was speculated to modulate PIA/eDNA-based biofilm formation by repressing *codY*. Therefore, YycF negatively modulates Cody for PIA/eDNA-based biofilms and also affects *ica* for PIA biofilms. Notably, the biofilm disassembly of the dual mutant (*codY* + AS*yycF*) was more obvious than that of *codY* + (Figures 2B, 4B). As *S. aureus* in biofilms is 1,000 times more tolerant to antibiotics and recalcitrance than planktonic cells, the susceptibility of the pathogen was reversible without the shelter of the biofilm (Shenkutie et al., 2020; Gimza and Cassat, 2021).

A potential mechanism of CodY limits the host damage of *S. aureus*, in which it transitions from a commensal bacterium to an invasive pathogen. The decreased CodY activity promotes a more invasive lifestyle of *S. aureus* (Waters et al., 2016). Similarly, our animal experimental results indicated that the ability of AS*codY* strains to invade was higher than that of *codY* + and they formed a larger abscess (Figure 6A). Similar with Montgomery et al., we found CodY can represses virulence *in vivo*. In Montgomery study, Cody as a global regulator can decrease expression of *agr* and *saeRS*, as well as the gene encoding the toxin alpha-hemolysin (hla). Also, Cody can restrain the expression of the lukF-PV gene, encoding part of the Panton-Valentine leukocidin (PVL) (Montgomery et al., 2012). By multiple pathways, Cody can mediate the virulence of USA300. In our study, CodY-mediated repression was focus on CodY/YycF interaction and biofilm formation. And our results indicated CodY can impede the pathogenicity of *S. aureus* by biofilm inhibition which has a potential role on immunoregulation. By histological examination, we observed that invasive AS*codY* stains could recruit more immune cell infiltration surrounding the infectious region than *codY* + strains (Figure 6B). Therefore, YycF could indirectly enhance bacterial aggregation by repressing CodY (Figure 7). According to this mechanism, our previous antisense *yycF* (AS*yycF*) is base-paired with *yycF* and downregulates *yycF* expression, which indicates therapeutic potential for infectious diseases (Wu et al., 2021a). One previous study indicated that CodY repression of *sae* expression (an exoprotein expression TCS SaeRS to secrete virulence factors) delays immune evasion and reduces immune cell death (Mlynek et al., 2018). In the present study, the AS*codY* group also had a higher apoptosis rate of macrophage cells than that of the *codY* + group (Figure 6C). However, in the *S. aureus* ATCC29213 group, the apoptosis rate was even higher than that in the AS*codY group*. This may be induced by the positive regulation of CodY on genes such as *fnbA* and *spa*, which encode the microbial surface components recognizing adhesive matrix molecule (MSCRAMM) proteins (Brinsmade, 2017). It could be speculated that CodY as a repressor of target can also positively regulate bacterial redox balance and protease induced biofilm formation (Shivers et al., 2006; Roux et al., 2014). In addition, the construction of AS*codY* including the introduction of an exogenous plasmid vector may interfere intracellular homeostasis (Senadheera et al., 2009; Lei et al., 2015). All those items will affect bacterial metabolism and indirectly interaction between AS*codY* and macrophages, which may attribute to a lower apoptosis rate in AS*codY* group instead of *S. aureus*.

## Conclusion

In summary, YycF binds to the promoter regions of *codY* and negatively regulates the expression of *codY*. By interacting with *codY*, YycF could modulate *S. aureus* biofilm formation *via* both eDNA-dependent and PIA-dependent pathways, which can be a significant target for anti-biofilms. CodY impedes pathogenicity and also has a role in immunoregulation. CodY not only impedes the pathogenicity but also has a role on immunoregulation. By interacting with CodY, YycF plays essential roles in host-pathogen interactions and pathogenesis. Thus, the current evidence may provide a supplementary strategy for managing biofilm infections.

## Data availability statement

The raw data supporting the conclusions of this article will be made available by the corresponding authors.

## Ethics statement

The animal study was reviewed and approved by West China Hospital Animal Welfare Committee (No. 20220606004).

## Author contributions

SW, BQ, LL, and GF: conceptualization (equal), data curation (equal), formal analysis (equal), validation (equal), writing—original draft and review, and editing (equal). SD, YL, and HZ: conceptualization (supporting), formal analysis (supporting), funding acquisition (lead), writing—review, and editing (equal). All authors contributed to the article and approved the submitted version.
