# Supplementary material for: CodY is modulated by YycF and affects biofilm formation in Staphylococcus aureus
Source: Front Microbiol. 2022 Oct 11;13:967567. doi: 10.3389/fmicb.2022.967567 (PMC9593060; doi:10.3389/fmicb.2022.967567)
Supplement: Supplementary file 1 [file Data_Sheet_1.docx]

CodY is modulated by YycF and affects biofilm formation in *Staphylococcus aureus*

Shizhou Wu^1#^, Boquan Qin^1#^, Shu Deng^2^, Yunjie Liu ^3^, Hui Zhang^1^, Lei Lei^4*^, Guoying Feng^5*^

^1^ Department of Orthopedics, West China Hospital, Sichuan University, Chengdu, China

^2^ Boston University Henry M Goldman School of Dental Medicine, Boston, MA 02101, USA;

^3^ West China School of Public Health, Sichuan University, Chengdu, China

^4^ Department of Preventive Dentistry, West China Hospital of Stomatology, Sichuan University, Chengdu, China

^5^ College of Electronics and Information Engineering, Sichuan University, Chengdu, Sichuan, 610064, China.

^#^Co-first authors:

wushizhou1990@wchscu.cn (S. Z. W);

^*^Correspondence:

guoing_feng@scu.edu.cn (G. Y. F); leilei@scu.edu.cn (L. L).

Keywords: Antisense; Biofilm formation; *Staphylococcus aureus*; YycFG; Cody

**Appendix**

***Methods and materials***

*Bacterial strains and biofilm growth conditions*

As previously described, *S. aureus* strain ATCC29213 was cultured in tryptic soy broth (TSB) at 37 °C and 5% CO_2_. Briefly, 500 μL of *S. aureus* suspension was inoculated into 10 mL fresh TSB medium to mid-logarithmic phase (optical density at 600 nm [OD_600_] = 0.5), and a log-phase suspension was prepared for further investigation. To detect the bacterial time growth curve, the bacterial strains were cultured at 37°C and growth curves were measured at OD_600_ each hour interval.

***PIA production*** ***using the anthrone-sulfuric method***

Overnight cultures of *S. aureus* strains were diluted 1:20 in fresh TSB, then grown to an OD_600nm_ of 0.3-0.5. The mid-log phase cells were then diluted 1:100 in fresh TSB. After incubation for 24h, the biofilms were collected by scraping using PBS buffer. The supernatant was separated for PIA measurement using the anthrone method. Six hundred microliters of anthrone reagent (200 mg anthrone dissolved in 100 mL concentrated sulfuric acid) were added to 200 μL of cell-free supernatant and mixed gently by inversion. The reaction mixtures were incubated at 95 °C for 6 min. The absorbance of each sample at 625 nm was monitored on a microplate reader (GeneCo, Hong Kong, China). The corresponding polysaccharide concentration was calculated according to the standard curve.

***eDNA production***

The biofilm matrix is formed by PIA, eDNA and proteins. Genomic DNA (gDNA) is released from bacterial cells into the environment by active secretion (Ibáñez de Aldecoa et al, 2017). The amount of eDNA in liquid cultures was measured directly by quantitative real-time PCR (Xu and Kreth, 2013). A 2-μl aliquot of cell-free culture supernatant was added for each reaction. Real-time PCR was carried out with a Light Cycler 480 II (Roche, CA, USA). For each real-time PCR reaction, 20 μL of a mixture containing 10 μL SYBR Premix Ex TaqII, 2.0 μL cell-free culture supernatant, 1μL 10 μM 16S rRNA Forward Primer, 1μL 10 μM 16S rRNA Reverse Primer, and 6.0μL deionized water was placed in each well. Conditions for Real-time PCR were as follows: 95°C, 3 min (initial denaturation), followed by 35 cycles of 95°C for 30 s (denaturation), 55°C for 30s (primer annealing), and 72°C for 30s (extension). Threshold cycle values (CT) were quantified and the expression of each gene was normalized relative to the expression of the *S. aureus* 16S rRNA gene, which was used as an internal reference. Data were calculated according to the 2^−ΔΔCT^ method.

***Western blotting analysis***

Overnight cultures of *S. aureus, codY+,* AS*codY* strains were diluted 1:20 in fresh TSB, then grown to an OD_600nm_ of 0.3-0.5. The harvested cells were washed and re-suspended in 300 µL cold phosphate buffered saline (PBS, pH 7.3). Cells were mechanically disrupted by ultrasonic disruption for three cycles of 20 sec. Clear supernatants were collected by centrifugation (13000 rpm, 2 min, 4°C) and protein concentrations were determined using a bicinchoninic acid (BCA) protein assay kit (BioRad, Hercules, CA, USA) according to the manufacturer’s instructions.

For western blotting analysis, equal amounts of protein (50 µg) were mixed with 2X SDS-PAGE Sample Loading Buffer (Beyotime Biotech, Shanghai, China) in boiling water for 10 min and loaded on 10% SDS-PAGE gels (Bio-Rad). Proteins were fractionated and then electrotransferred to polyvinylidene fluoride (PVDF) membranes (Biosharp Biotech, Shanghai, China). Membranes were blocked in TSBT buffer (25mM Tris, 140mM NaCl, 3mM KCl, 0.1% Tween 20) containing 5% w/v nonfat dry milk at room temperature for 1 h. The membranes were incubated with purified CodY-specific antibodies (1:1000, AbMax Biotechnology, Beijing, China) overnight at 4°C, washed in TSBT buffer, and incubated with horse-radish peroxidase (HRP)-conjugated goat anti-mouse secondary antibody (1:10000) for 2 h at room temperature. Protein immunoreactive bands were visualized using an Immobilon Western Chemiluminescent kit (Millipore, Billerica, MA, USA). The ChemiDoc MP Imaging system (Bio-Rad) was used to detect the signal density of the protein bands.

**References**

Ibáñez de Aldecoa AL, Zafra O, González-Pastor JE. 2017. Mechanisms and regulation of extracellular DNA release and its biological roles in microbial communities. Front Microbiol 8:1390.

Xu Y, Kreth J. 2013. Role of LytF and AtlS in eDNA release by Streptococcus gordonii. PLoS One. 2013 Apr 24;8(4):e62339.

The anti-sense sequence of *codY*

TTATTTACTTTTTTCTAATTCATCTAAGAATTTTTCTTTTTTAACTTTAATGAAAGTACCTTTCATTCCTAAAGAACGTGATTCAATTACACCAGCACTTTCTAATTTACGTAGTGCATTTACAATTACAGATCTAGTAATACCAACTCTATCTGCAACTTTTGATGCGATTAATAGGCCTTCCGTACCGCCAAGTTCTTCAAAGATATGTTCAATCGCTTCTTTTTCAGAATAAGATAATGAATTAATTGCCATTGTAATAGCAGCTTTATCGCGCGCTTCTTTTTCTACTTCACTATGCTTCTCACGTAAGATTTCCATACCAATAACTGTAGCAGCATATTCACCTAGTACCAAATCATTTTCATTAAAATCATCATGTACTCGACCAAGTACTAATGTACCTAATCTTTCCCCTCCACCTAAAATTGGGAAGATAGTTGTACGACTATCTATGAATAATTCTCTGTTTTCAGGTGGGAATACTGTTAATACATTGTCGATATCAATATTTGATTCTGTTTGTTTAACTTCCATTAATCGTTCTGTATATTCACTTGGAATATGTCTTTCTTCCAACATTTGAATAATTCTTTGACTTTTTAATAATTCATTTAGACTCGATCCTAAAATTTTACCTCGACGCGATACAATAAATACATTTGTTACAGTTACGCTACTAATCGTTTGTGCTACATCTTTAAAATCAACCGCAATACCTTTGTGTTTTTGAAGTAACGTGTTTAACTCTCTCGTTTTAGATAATAAGCTCAT


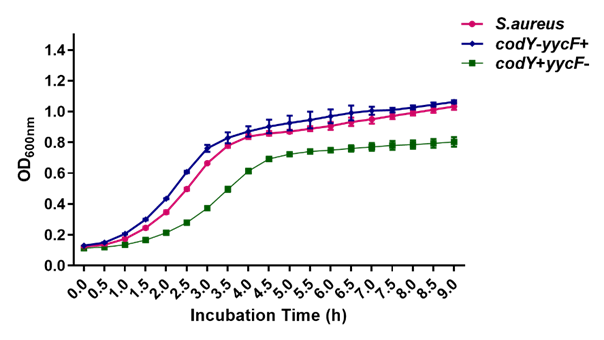


**Figure S1**. Bacterial time growth curve for dual mutants


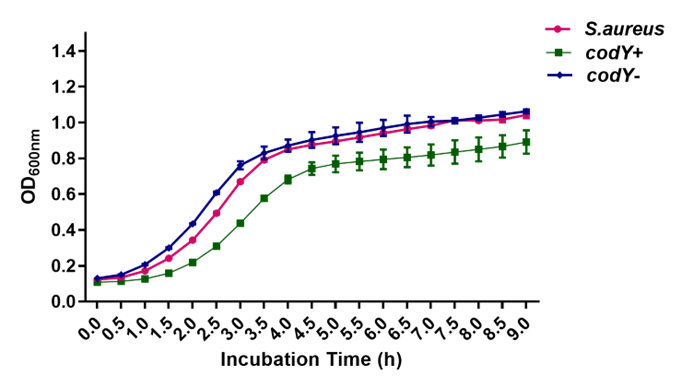


**Figure S2**. Bacterial time growth curve for *codY* mutants


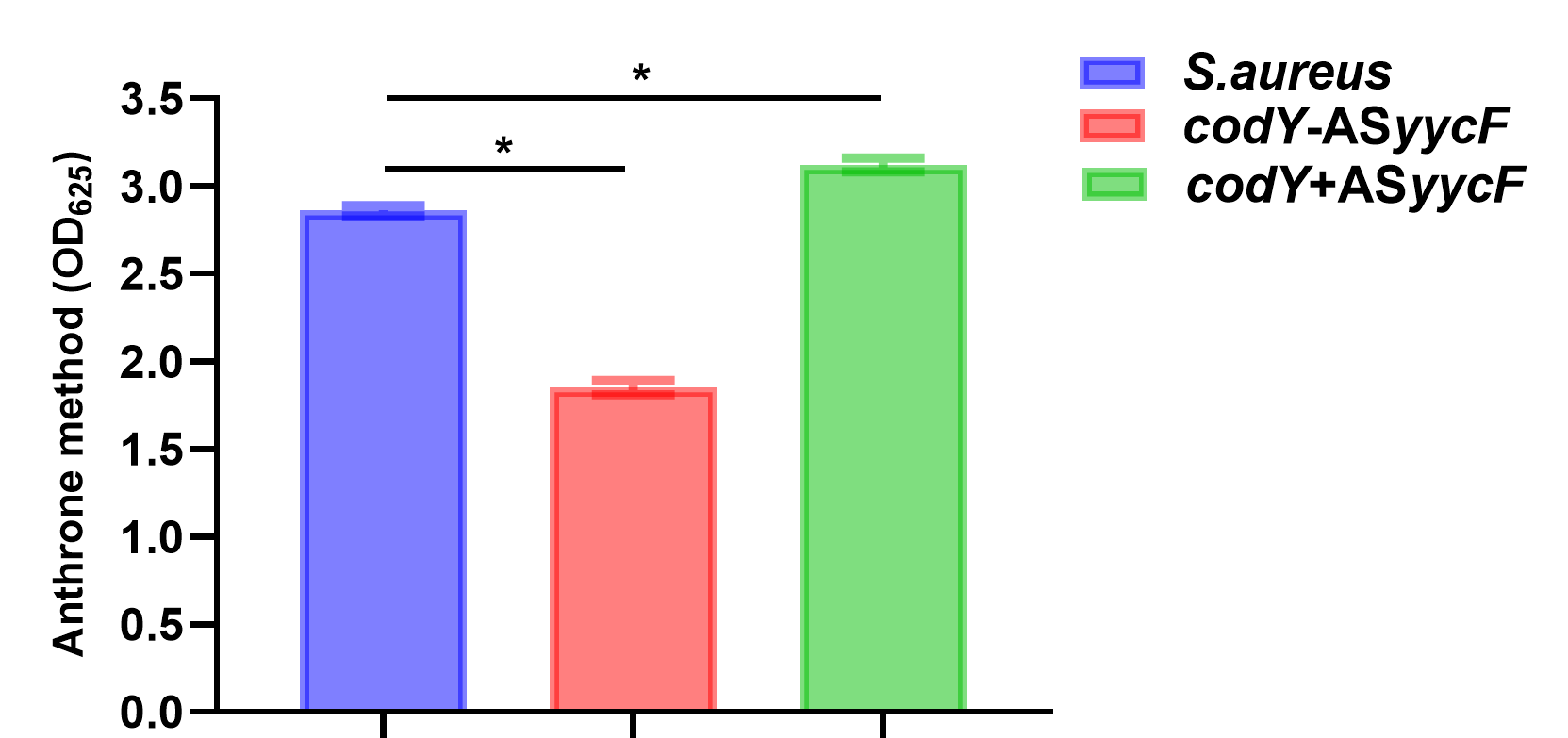


**Figure S3**. PIA production for dual mutants (^*^*P*<0.05)


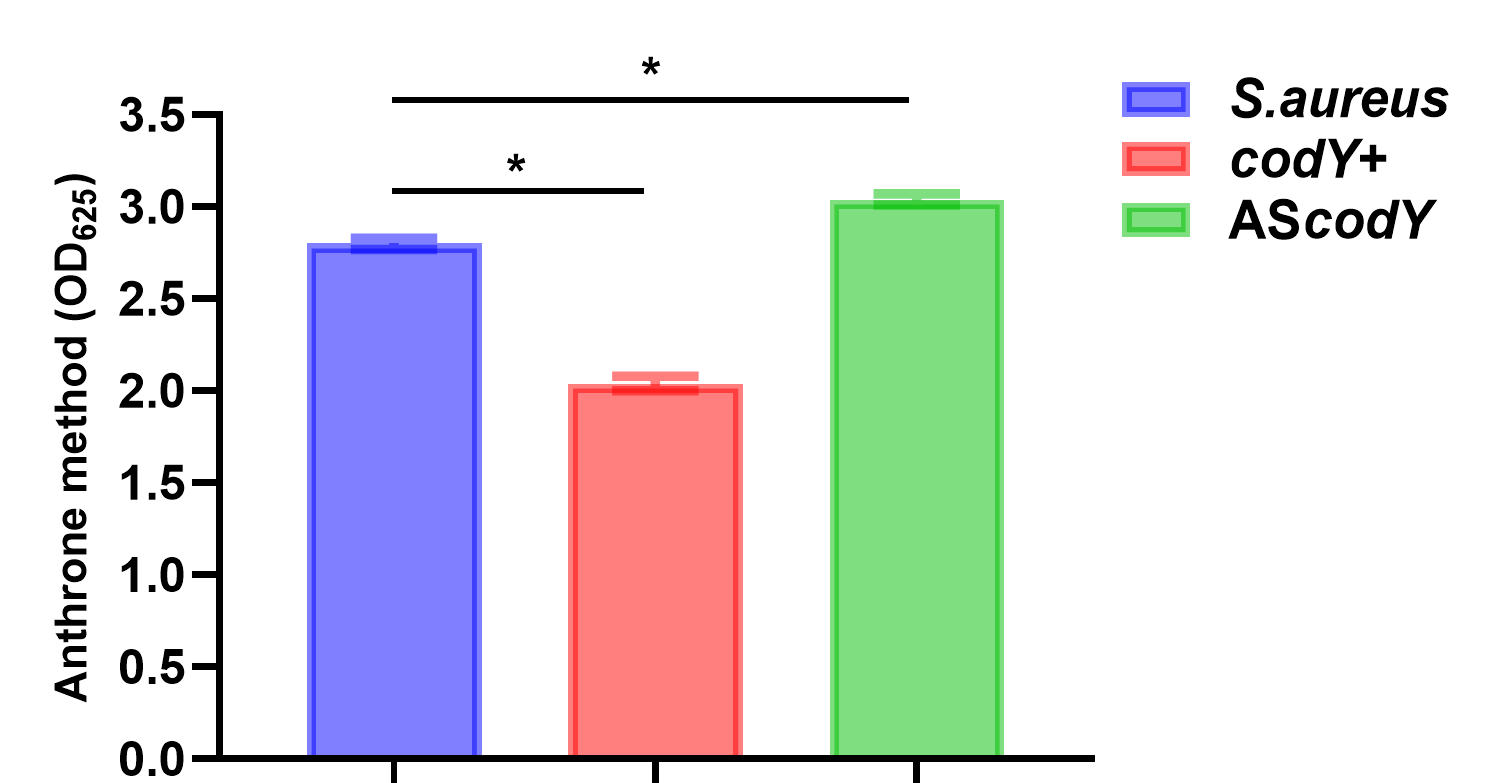


**Figure S4**. PIA production for *codY* mutants (^*^*P*<0.05)


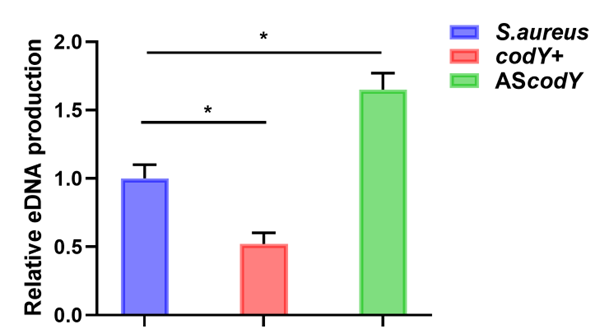


**Figure S5**. eDNA production for *codY* mutants (^*^*P*<0.05)


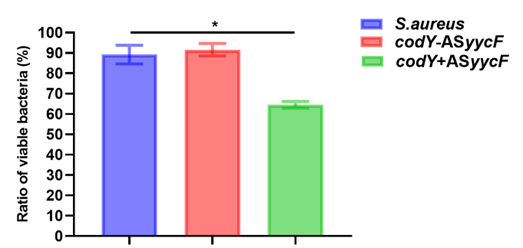


**Figure S6.** The ratio of viable bacteria for dual mutants (**P*<0.05)


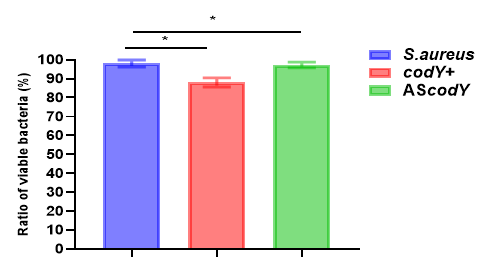


**Figure S7.** The ratio of viable bacteria for *codY* mutants (**P*<0.05)


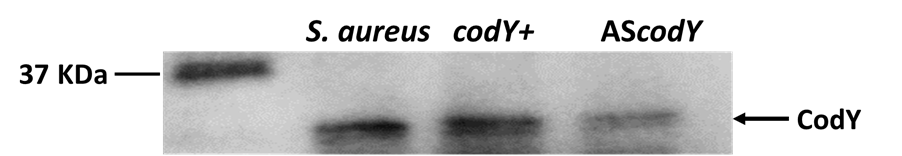


**Figure S8**. Western blotting analysis for CodY production
